# Associations between Adolescent Psychosocial Factors and Disengagement from Education and Employment in Young Adulthood among Individuals with Common Mental Health Problems

**DOI:** 10.1007/s10964-022-01592-7

**Published:** 2022-03-11

**Authors:** Sümeyra N. Tayfur, Susan Prior, Anusua Singh Roy, Donald Maciver, Kirsty Forsyth, Linda Irvine Fitzpatrick

**Affiliations:** 1 School of Health Sciences, Queen Margaret University, Edinburgh, UK; 2 Strategic Programme Manager, Mental Health and Wellbeing, City of Edinburgh Health and Social Care Partnership, Edinburgh, UK

**Keywords:** Mental health, Psychosocial, Adolescence, Young adulthood, Education, Employment, NEET, Longitudinal

## Abstract

Transition to adulthood can be a challenging developmental task for adolescents with common mental health problems and is linked to adverse outcomes such as ‘not in education, employment or training’ (NEET). This study investigated longitudinal associations between adolescent psychosocial factors (e.g., self-esteem, aspirations, bullying, physical activity) and later NEET status among individuals with common mental health problems (i.e., depression and anxiety). A secondary data analysis of the Next Steps cohort study was completed using waves 2 and 8. Psychosocial factors, mental health, and background characteristics were captured when participants were aged 15–16 years (wave 2) while still in compulsory education. The 12-item General Health Questionnaire was used to identify adolescents with common mental health problems. The study population consisted of 2224 participants (females 66.8%) of which 1473 (66.2%) were aged 15 years and 751 (33.8%) were aged 16 years in wave 2. The outcome was NEET status at ages 25–26 years (wave 8). The results showed that after adjusting for background characteristics, adolescent self-esteem, locus of control, bullying, physical activity, job aspirations, and attitudes to school predicted NEET status. Educational aspirations, substance use, and behavioural problems were not significantly associated with NEET status. These findings provide new insights into the role of adolescent psychosocial factors in the context of education and employment outcomes for youth at risk and highlight the necessity of targeted mental health support to improve life chances.

## Introduction

Adolescents with common mental disorders struggle with successfully transitioning into adulthood and experience poor health, social, educational, and economic outcomes (Wagner & Newman, 2012). They are more likely to get disengaged from education and employment as young adults, a status referred to as NEET (not in education, employment, or training) (e.g., Witt et al., 2019). More importantly, those with subclinical depression and anxiety experience equal functional impairment as those with clinical levels (Wesselhoeft et al., 2013, Balázs et al., 2013). They experience long-term consequences in the same areas including becoming NEET (Clarke & Lovewell, 2021). However, there is a significant care gap for this group of young people as the majority do not access mental health services and receive early intervention (Soneson et al., 2022). There is a need to focus on adolescents with such common mental health problems before they transition from compulsory education (e.g., Cornaglia et al., 2015). A comprehensive investigation of what factors are associated with later disengagement from education and employment is lacking for this population in the extant literature. This would provide important insights for preventive strategies during secondary schooling. The current study focused on longitudinal associations between adolescent psychosocial factors and NEET status in young adulthood among individuals with common mental health problems.

Adolescents with mental health problems pose a serious and growing issue as a public health challenge. Among secondary school-aged adolescents in England, one in six (17.6%) is identified with a probable mental disorder, an increase from 13.6% in 2017 (Clarke et al., 2020). Depression and anxiety are the most common mental disorders experienced by young people (Clarke et al., 2020). Prevalence is higher in female adolescents than males (Balázs et al., 2013). Adolescents are least likely to seek treatment compared to any other age group and only about half access services in England (Soneson et al., 2022). One of the reasons is under-identification of needs (Soneson et al., 2022). They are poorly understood, go unrecognized by the school services, and lack the support they need at an early stage which leads to more severe psychopathology (Baksheev et al., 2011). Long-term consequences of such problems regarding impaired role functioning have gained increasing recognition and the impact appears to be substantial and greater than that associated with physical disorders (e.g., Mojtabai et al., 2015).

Adolescents with common mental health problems face unique challenges when transitioning from school to further education and employment. They experience adverse labour market outcomes in adulthood such as reduced employment and earnings (e.g., Fletcher, 2013). They are also more likely to become NEET in young adulthood (e.g., Cornaglia et al., 2015; Witt et al., 2019). In the UK, recent figures show that the total number of people aged 18 to 24 years who are in NEET status is 744,000 which represents 13.8% of young adults in the same age range (Office for National Statistics, 2021). There are consequences to being NEET such as health, societal and economic burden to the government and poor health, prolonged unemployment, low wages, and social exclusion to the individual (Arnold & Baker, 2013).

Exploration of risk factors for becoming disengaged from education and employment is necessary to identify targeted preventive strategies for adolescents with common mental health problems during compulsory education. This is crucial not just because of the associated long-term consequences, but also because intervention is difficult once they become disengaged and there is a high recurrence rate (Arnold & Baker, 2013). A young person who enters NEET category once is 7.9 times more likely to become disengaged again (Arnold & Baker, 2013). A simple measure such as the 12-item General Health Questionnaire (GHQ-12) is applicable in secondary school settings to identify those with common mental health problems (Baksheev et al., 2011). It is also useful for predicting who is at risk of getting disengaged from education and employment (Cornaglia et al., 2015). Early identification through screening at schools using GHQ-12 rather than relying on help-seeking would aid in preventing development of severe psychopathology and lead to better long-term outcomes (Baksheev et al., 2011).

Research has mainly focused on background characteristics as risk factors for NEET. Socioeconomic disadvantage, single-parent household, being a young carer or teenage parent, and educational attainment are some of the key risk factors (Dorsett & Lucchino, 2014, Pitkänen et al., 2021). There are a number of additional important psychosocial risk factors at play. Psychosocial is an umbrella term that describes the intersection and interaction of social, cultural, and environmental influences on the mind and behaviour (American Psychological Association, 2020). A recent systematic review identified that various domains of adolescent psychosocial factors such as behavioural problems and bullying are associated with education and employment status in young adulthood (Tayfur et al., 2021). Attitudes to school has also been associated with later education and employment status (Duckworth & Schoon, 2012, Spengler et al., 2018). Since no risk factor occurs in isolation in shaping a developmental outcome (Duckworth & Schoon, 2012), it is important to identify the independent and relative role of these psychosocial factors with disengagement from education and employment for vulnerable youth.

## Current Study

Adolescents with common mental health problems are poorly understood in their transitions from school to further education and employment. The current study aims to identify longitudinal associations between adolescent psychosocial factors and subsequent NEET status among this population in a nationally representative sample of secondary school students. More specifically, the study focuses on behavioural problems, bullying, substance use, self-esteem, locus of control, physical activity, attitudes to school, and educational and job aspirations. Two research questions are addressed. Are there any significant differences in the background characteristics of a nationally representative sample of adolescents with common mental health problems who become engaged in or disengaged from education and employment in young adulthood (Research Question 1)? What are the independent and relative associations of adolescent psychosocial factors with disengagement from education and employment in young adulthood after controlling for background characteristics of a nationally representative sample of adolescents with common mental health problems (Research Question 2)?

## Methods

### Study Population

The Next Steps cohort study (previously known as the Longitudinal Study of Young People in England) is a nationally representative eight-wave survey of youth in England which provides an excellent opportunity to examine prospective associations of a broad range of psychosocial factors and education and employment outcomes in a relatively large national sample. The Next Steps has surveyed young people from 892 schools annually since 2004 starting with respondents of age about 14 years (*n* = 15,770) to age 25–26 years (*n* = 7,707) in 2015. The data from waves 2 (age 15) and 8 (age 25), collected in 2005 and 2015, respectively are used in this study. wave 2 was selected because participants were aged 15–16, therefore, preparing to transition from compulsory education. A 10-year follow-up between the selected waves eliminates the turbulent phase of transition into adulthood, a demanding process with many dimensions, in order to investigate developmental outcomes including educational and occupational engagements (Scales et al., 2016). Appropriate weights are supplied within the dataset for the analyses to account for the complex survey design and attrition. Detailed methodology regarding the complex design features (two-stage probability proportional to size sampling procedure with disproportionate stratification) and ethical approval of the dataset can be reached from the available documentation of the Centre for Longitudinal Studies and the dataset can be accessed through UK Data Service (University College London, UCL Institute of Education, Centre for Longitudinal Studies (2021)).

**Fig. 1:**
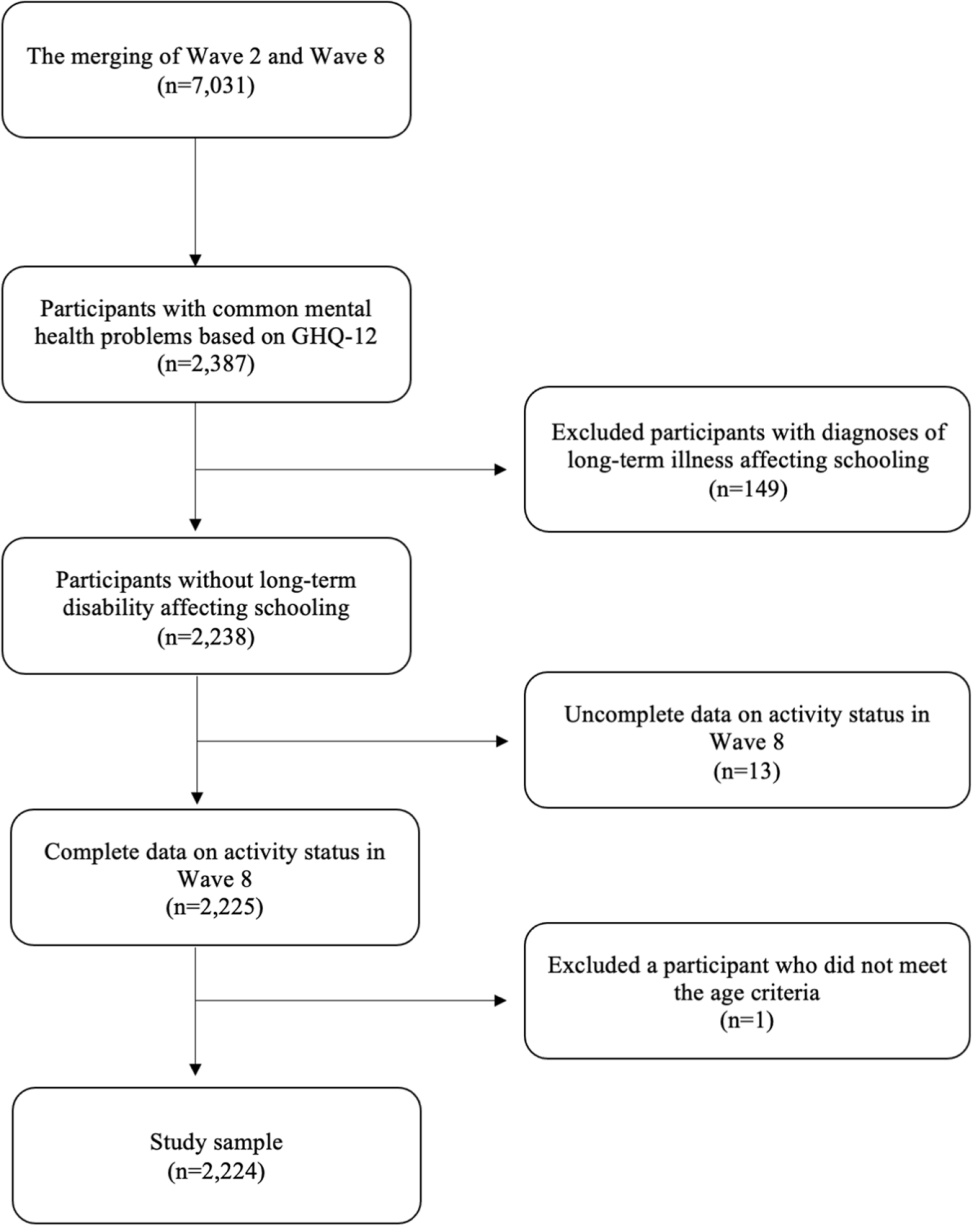
Sampling of the data

The sample is restricted to those with common mental health problems using the GHQ-12 available in wave 2. GHQ-12 is widely used as a nonspecific measure of common mental disorders (Lundin et al., 2016). It is used in public health surveys including a valid use among adolescent populations (e.g., Baksheev et al., 2011). It is scored between 0 and 12 and the recommended optimal cut-off thresholds are either ≥ 2 or ≥ 3 (Lundin et al., 2016). Both perform equally well at distinguishing cases from non-cases (Lundin et al., 2016). The former was selected as there was no need to be stringent given the literature. Figure 1 shows the selection of the data for the current study. The merging of wave 2 and wave 8 led to a sample of 7,031 participants who responded in both waves. Then, the sample was disaggregated using the GHQ-12 and ≥ 2 as the cut-off point which reduced the sample size to 2,387. Long-term illness/disability which affected the participants’ schooling was measured in wave 2. This was indicative of serious disorders that interfered with schooling likely requiring special education; therefore, these individuals were excluded from the sample which further reduced the sample size to 2,238. It was expected that all participants have the current activity status available in wave 8 which is the dependent variable in the current study. Those who did not have this information available were excluded which led to a total of 2,225 participants. There was one participant aged 27 in wave 8 which did not fit the age criteria of the current study because young adulthood is typically defined as ages between 18–26 so that case was excluded (Arnett, 2000). These steps led to a final sample size of 2,224 in the current study. While 66.2% of participants (*n* = 1473) were aged 15 years, 33.8% were 16 years old (*n* = 751) in wave 2. However, they were all in the same compulsory school year. None of the participants was in care and all were living in private households.

### Measures

#### Psychosocial factors

##### Self-esteem

It was defined on participant’s response to how much a young person has been thinking of himself/herself as a worthless person recently (Mendolia & Walker, 2015). This item was measured on a 4-point Likert scale. ‘Not at all’ was the reference category in the analyses.

##### Locus of control

It was defined based on whether a participant agrees that if he/she works hard at something, then he/she would usually succeed. If the participant agreed, then it was coded as ‘internal’ (Ng-Knight & Schoon, 2017).

##### Educational Aspirations

It was measured by a young person’s intentions after year 11 with categories of staying on in full-time education reflecting high aspirations or leaving full-time education as low educational aspirations. If the response was ‘don’t know’, it was coded as ‘uncertain’ (Gutman & Schoon, 2018).

##### Job aspirations

The item available in the dataset includes whether a participant had a paid job during term time. If the respondent agreed, then it was coded as a ‘yes’. Those engaging in work during school are expected to have higher aspirations towards a career track after compulsory education (Baert et al., 2017).

##### Physical activity

It was defined based on self-reporting of frequency of doing sports such as football or swimming. While being active most days was coded as ‘high’ averaging about once a week was coded as ‘moderate’ and those who responded as ‘hardly ever’ and ‘never’ were categorized as ‘low or none’ parallel to the recommendations by World Health Organization (World Health Organization, 2020).

##### Attitudes to school

It was measured by a participant’s response to twelve items related to feelings about school. It sums answers to those attitudinal questions and ranges from 0–48 with higher scores indicating more positive attitudes (Duckworth & Schoon, 2012). The Cronbach’s alpha is 0.82. Confirmatory factor analysis showed that the twelve items all loaded satisfactorily on a single factor (all std. loadings > 0.3, *p* < 0.001).

##### Bullying

This is a derived variable made available within wave 2 based on any self-reporting of a participant being bullied in any way since the start of the cohort study. If the respondent reported being bullied, it was coded as a ‘yes’ consistent with previous studies (Strøm et al., 2013).

##### Substance use

Adolescents were considered to engage in harmful substance use if they agreed to at least two of the three items based on whether they had a proper alcoholic drink, smoked cigarettes, or tried cannabis in the last year. Similar measures have been used in previous research (Goldman‐Mellor et al., (2016)).

##### Behavioural problems

It was defined based on whether a participant had taken part in public disturbance, shoplifting, vandalizing public property, or graffitiing in the last year. If the participant agreed to at least one of the four items, it was coded as a ‘yes’. These items have been included in the Edinburgh Study of Youth Transitions in Crime (Smith & McVie, 2003).

##### NEET status

It was based on participant’s main activity status reported at age 25–26 years. Young adults who were NEET were identified as those who were unemployed and not enroled in school, training, an apprenticeship, or postsecondary education (Office for National Statistics, 2021). Of the sample, 12.4% were in NEET status at age 25–26 years (*n* = 276). The majority of respondents classified as NEET were unemployed (43.1%). A further 41.7% reported their main activity as looking after the home. The remaining 15.2% were doing voluntary (unpaid) work, travelling or were NEET due to illness or disability.

#### Control variables

##### Sex

Sex was self-reported by the participants. It was coded as ‘female’ and ‘male’.

##### Socioeconomic status

It was defined based on the English Index of Multiple Deprivation (IMD) rounded scores available in wave 2. IMD categorizes areas in England based on seven domains of deprivation including income, employment, health, education and training, housing and services, living environment, and crime. It ranges between 1 and 86 and as the score increases, deprivation also increases.

##### Ethnicity

It was reported as respondents self-identifying into eight categories: White, Mixed, Indian, Pakistani, Bangladeshi, Black Caribbean, Black African and other. Due to a small proportion of respondents in some of these categories, it was dichotomized as ‘White’ and ‘Other’.

##### Caring responsibility

It was measured by self-reporting of whether a participant was looking after someone in the household with a binary variable as ‘yes’ and ‘no’. Potential young carers or teenage parents were expected to fall into this category.

##### Family composition

It was based on parent reports and derived into five categories: Married, cohabiting, lone father, lone mother, and no parent. Due to small proportion, these categories were combined as ‘married/cohabiting’ and ‘single/no parent’.

### Statistical Analyses

The associations between background characteristics and NEET status were examined by using the Pearson Chi-square test and are presented as the Rao-Scott adjusted statistic. For the correlations between dichotomous categorical variables, the Chi-square test is used, and the phi coefficient (same as Cramer’s v) is reported to show the strength of correlations. If more than two categories were present, then the Cramer’s v is reported since the phi-coefficient is only applied to two dimensional tables. For the correlations between continuous variables, the Spearman test is carried out and the Spearman’s rho is reported whereas for a correlation between a categorical and a continuous variable, the Kruskal–Wallis test was carried out and the Epsilon-squared is reported. Psychosocial factors and subsequent NEET status were examined with binary logistic regression analyses. Results for the logistic regression analyses are presented as odds ratios of being in NEET status at follow-up. The multivariable logistic regression model is adjusted for sex, ethnicity, socioeconomic status, caring responsibility, and family composition.

All analyses were conducted using the R software (R Core Team, 2021). All analyses were weighted to account for complex sampling design and attrition. *Survey* package in R was mainly used for this purpose (Lumley, 2020). Less than 6% data was missing for each variable and therefore complete case analysis was carried out. However, this has led to reduction in the sample size from 2224 to 1,849 in the multivariable logistic regression model. When more than 10% of cases have missing data, multiple imputation is recommended (Langkamp et al., 2010). It is not possible to establish from the observed data alone whether the pattern of missingness is ‘missing at random’ (MAR) or ‘missing not at random’ (MNAR) (White et al., 2011). Missing data analysis suggested the pattern is most likely MAR and certainly not ‘missing completely at random’ in the current study. Since this is also secondary data, a distinction cannot be made between MAR and MNAR. However, multiple imputation is a strong technique that is effective with MAR but also MNAR data, especially if less than 30–50% missing data are to be imputed (White el al., 2011). Therefore, multiple imputation was performed for the multivariable model to compare with the results from casewise deletion which is the default in R. Since 20 imputations are recommended for 10% to 30% missingness, 20 imputations were implemented in the current study (Graham et al., 2007).

## Results

Table 1 shows the general characteristics of the sample stratified by young adult NEET status. Of the current sample, 66.8% were female (*n* = 1486) and 68.9% of participants were White (*n* = 1,533). Only 5.7% had a caring responsibility within the household (*n* = 126) and mean socioeconomic status of the participants were 22.8. In terms of household, the majority came from English speaking (89.4%) and married or cohabiting families (78.4%). It is found that young adults who were in NEET status significantly differed in respect to some characteristics during adolescence. Differences were established in relation to sex (F_adj_ = 7.02, *p* = 0.008); caring responsibility (F_adj_ = 7.66, *p* = 0.006); family composition (F_adj_ = 15.25, *p* < 0.001), and socioeconomic status (t = 7.3, *p* < 0.001). Ethnicity (F_adj_ = 0.594, *p* = 0.441) was not significant.

**Table 1:** Baseline characteristics of the sample stratified by NEET status at age 25–26 years

		Total (*n* = 2224)		In education, employment or training (EET) (*n* = 1948)		Not in education, employment or training (NEET) (*n* = 276)		
		*n*^a^	%	*n*	%	*n*	%	χ^2^ test
**Sex**	Female	1486	(66.8)	1282	(65.8)	204	(73.9)	*p* = 0.008
	Male	738	(33.2)	666	(34.2)	72	(26.1)	
**Ethnicity**^b^	White	1533	(68.9)	1347	(69.2)	186	(67.6)	*p* = 0.441
	Other	688	(30.9)	599	(30.8)	89	(32.4)	
**Caring responsibility**	Yes	126	(5.7)	99	(5.1)	27	(9.8)	*p* = 0.006
	No	2093	(94.1)	1844	(94.9)	249	(90.2)	
	No	234	(10.5)	191	(9.8)	43	(15.6)	
**Family composition**	Married/ cohabiting	1743	(78.4)	1554	(80.7)	189	(70.8)	*p* < 0.001
	Single/no parent	449	(20.2)	371	(19.3)	78	(29.2)	
		*n*^a^	SD	Mean	SD	Mean	SD	Kruskal-Wallis test
**Socioeconomic status**	(Range: 1-86)	22.8	16.9	21.8	16.3	30.1	19.1	*p* < 0.001

*Note*. All analyses are adjusted for complex survey design and attrition

^a^Total cell counts for each variable may not add up to 2224 and percentages to 100% due to missingness

^b^Other ethnicities include: Indian, Pakistani, Bangladeshi, Black Caribbean, Black African, Mixed, and Other

Correlations between variables are reported in Table 2. The interpretation for the phi coefficient is that between 0.10–0.30 are small, 0.30–0.50 are medium and ≥ 0.50 are large and Cramer’s v for at least 3 categories in either row or column are interpreted as 0.07–0.20 small, 0.20–0.35 medium and ≥0.35 large (Mangiafico, 2016). As for the Epsilon-squared, between 0.01–0.08 are small, 0.08–0.26 are medium and ≥ 0.26 large (Mangiafico, 2016). None of the significant correlations were found to be of large size. Since the goal is to avoid inclusion of explanatory variables correlated at *r* ≥ 0.8 (large), the significant correlations were in a range of tolerable inter-correlation for logistic regression analysis (Field, 2013).

**Table 2:** Correlations between variables

Items	1	2	3	4	5	6	7	8	9	10	11	12	13	14
1. Self-esteem														
2. Locus of control	**0.13**^b^													
3. Substance use	0.**16**^b^	**0.06**^a^												
4. Bullying	**0.13**^b^	**0.06**^a^	**0.08**^a^											
5. Behavioural problems	**0.12**^b^	**0.08**^a^	**0.35**^a^	**0.08**^a^										
6. Educational aspirations	0.06^b^	0.04^a^	**0.13**^a^	0.03^a^	**0.15**^a^									
7. Attitudes to school	**0.09**^c^	**0.10**^c^	**0.11**^c^	**0.04**^c^	**0.09**^c^	**0.12**^c^								
8. Job aspirations	0.05^b^	0.03^a^	**0.11**^a^	0.03^a^	0.002^a^	0.04^a^	0.03^c^							
9. Physical activity	**0.07**^b^	0.01^c^	**0.08**^b^	0.06^b^	0.05^b^	0.03^b^	**0.04**^c^	**0.08**^b^						
10. Sex	**0.16**^b^	**0.06**^a^	**0.11**^a^	0.002^a^	**0.10**^a^	**0.14**^a^	0.03^c^	0.02^a^	**0.22**^a^					
11. Ethnicity	0.04^b^	0.009^a^	**0.19**^a^	**0.09**^a^	0.003^a^	**0.11**^a^	**0.06**^c^	**0.16**^a^	0.04^a^	0.02^a^				
12. Socioeconomic status	**0.05**^c^	0.04^c^	0.07^c^	0.05^c^	**0.07**^c^	**0.07**^c^	-0.05^d^	**0.10**^c^	**0.05**^c^	0.02^c^	**0.19**^c^			
13. Family composition	**0.10**^b^	0.02^a^	0.05^a^	0.03^a^	**0.09**^a^	0.03^a^	**0.05**^c^	0.02^a^	**0.07**^a^	0.02^a^	**0.06**^a^	**0.10**^c^		
14. Caring responsibility	0.03^b^	0.02^a^	0.02^a^	**0.06**^a^	0.04^a^	0.003^a^	0.03^c^	0.008^a^	0.04^a^	0.04^a^	0.05^a^	**0.11**^c^	0.05^a^	

a = Phi coefficient; b = Cramer’s v; c = Epsilon-squared; d = Spearman’s rho

Bold numbers indicate statistical significance at the 5% level

The univariable logistic regression results are presented in Table 3. Results showed that lower self-esteem (COR 2.89, 95% CI 1.89–4.42), external locus of control (COR 1.76, 95% CI 1.01–3.06), behavioural problems (1.58, 95% CI 1.13–2.20), low/uncertain educational aspirations (COR 1.64, 95% CI 1.08–2.50), no job aspirations (COR 1.83, 95% CI 1.24–2.72), and low/no physical activity (COR 2.60, 95% CI 1.68–4.04) in adolescence significantly increased the likelihood of being NEET as young adults whereas positive attitudes toward school (COR 0.96, 95% CI 0.94–0.98) and not being bullied (COR 0.54, 95% CI 0.36–0.79) significantly decreased odds for being NEET at age 25–26 years. Substance use was found to be insignificant at 5% level. Regarding the interpretation of the size of the odds ratios, 1.68, 3.47, and 6.71 are equivalent to Cohen’s *d* = 0.2 (small), 0.5 (medium), and 0.8 (large), respectively (Chen et al., 2010). Therefore, effect sizes were small for behavioural problems, educational aspirations, attitudes to school, and bullying ranging from 0.54 to 1.64. Moreover, effect sizes were medium for self-esteem, locus of control, job aspirations and physical activity odds ratios ranging from 1.76 to 2.89 considering that the range is between 1.68 and 3.47 (Chen et al., 2010).

**Table 3:** Logistic regression analysis of adolescent psychosocial factors and young adult NEET status

	Univariable analysis				Multivariable analysis^a^		
	*n*	COR	95% CI	*p* value	AOR	95% CI	*p* value
**Self-esteem** (Much more than usual vs. not at all)	2161	2.89	[1.89–4.42]	<0.001	1.75	[1.06–2.89]	0.029
**Locus of control** (External vs. internal)	2182	1.76	[1.01–3.06]	0.045	1.93	[1.08–3.45]	0.027
**Substance use** (Yes vs. no)	2100	1.35	[0.98–1.86]	0.064	1.02	[0.69–1.51]	0.923
**Behavioural problems** (Yes vs. no)	2133	1.58	[1.13–2.20]	0.008	0.92	[0.61–1.38]	0.676
**Bullying** (No vs. yes)	2125	0.54	[0.36–0.79]	0.002	0.57	[0.36–0.90]	0.017
**Educational aspirations** (Low/uncertain vs. high)	2224	1.64	[1.08–2.50]	0.021	1.34	[0.80–2.25]	0.262
**Job aspirations** (No vs. yes)	2221	1.83	[1.24–2.72]	0.003	1.70	[1.09–2.65]	0.019
**Attitudes to school** (Range: 0-48)	2224	0.96	[0.94–0.98]	<0.001	0.97	[0.95–0.99]	0.009
**Physical activity** (Low/none vs. high)	2223	2.60	[1.68–4.04]	<0.001	1.98	[1.16–3.38]	0.013

All analyses are adjusted for complex survey design and attrition. ^a^ *n* = 1849. *COR* crude odds ratio; *AOR* adjusted odds ratio; *CI* confidence interval. The multivariable model additionally controlled for sex, ethnicity, socioeconomic status, caring responsibility, and family composition. Statistically significant covariates were socioeconomic status (AOR 1.03, 95% CI 1.02–1.04), family composition (AOR 1.87, 95% CI 1.28–2.73), and caring responsibility (AOR 0.53, 95% CI 0.29–0.97)

To examine whether the observed differences could be explained by variations in sex, ethnicity, socioeconomic status, caring responsibility and family composition, the analysis was adjusted for these covariates. The observed differences did not change except for educational aspirations and behavioural problems. As seen in Table 3, the multivariable logistic regression model showed that after adjustment for background characteristics, lower self-esteem (AOR 1.75, 95% CI 1.06–2.89), external locus of control (AOR 1.93, 95% CI 1.08–3.45), no job aspirations (AOR 1.70, 95% CI 1.09–2.65), and low to no physical activity (AOR 1.98, 95% CI 1.16–3.38) in adolescence significantly increased the likelihood of being NEET as young adults whereas positive attitudes toward school (AOR 0.97, 95% CI 0.95-0.99) and not being bullied (AOR 0.57, 95% CI 0.36–0.90) significantly decreased odds for being NEET at follow-up. While the effect size for bullying and attitudes to school was small, it was medium for self-esteem, locus of control, job aspirations, and physical activity (Chen et al., 2010). The goodness of fit statistics, discriminatory power, and assumptions were tested to establish the validity of the logistic regression model.

The multiple imputation results are shown in Table 4. After controlling for sex, ethnicity, socioeconomic status, caring responsibility, and family composition, lower self-esteem (AOR 1.76, 95% CI 1.11–2.79), no job aspirations (AOR 1.58, 95% CI 1.05–2.37), and low to no physical activity (AOR 1.90, 95% CI 1.18–3.04) in adolescence significantly increased the likelihood of being NEET as young adults whereas positive attitudes toward school (AOR 0.97, 95% CI 0.95–0.99) and not being bullied (AOR 0.63, 95% CI 0.41–0.96) significantly decreased odds for being NEET at follow-up. The magnitude of the associations was found to be similar to those of complete case analysis. It was small for job aspirations, bullying, and attitudes to school since up to 1.68 is considered small (Chen et al., 2010). The magnitude was medium for self-esteem and physical activity since the range is between 1.68 and 3.47 (Chen et al., 2010). The only statistically significant difference between the complete case analysis and the multiple imputation was observed for locus of control. Although it was significant at 5% level in the former, the multiple imputation suggested otherwise (Table 4).

**Table 4:** Multiple imputation results for the multivariable logistic regression analysis

	AOR	95% CI	*p* value
**Self-esteem** (Much more than usual vs. not at all)	1.76	[1.11–2.79]	0.016
**Locus of control** (External vs. internal)	1.37	[0.76–2.46]	0.291
**Substance use** (Yes vs. no)	0.91	[0.62–1.33]	0.630
**Behavioural problems** (Yes vs. no)	1.14	[0.79–1.66]	0.481
**Bullying** (No vs. yes)	0.63	[0.41–0.96]	0.033
**Educational aspirations** (Low/uncertain vs. high)	1.27	[0.80–2.02]	0.305
**Job aspirations** (No vs. yes)	1.58	[1.05–2.37]	0.026
**Attitudes to school** (Range: 0-48)	0.97	[0.95–0.99]	0.036
**Physical activity** (Low/none vs high)	1.90	[1.18–3.04]	0.008

All analyses are adjusted for complex survey design and attrition. *AOR* adjusted odds ratio; *CI* confidence interval, *n* = 2224. The model additionally controlled for sex, ethnicity, socioeconomic status, caring responsibility, and family composition. Statistically significant covariates were socioeconomic status (AOR 1.03, 95% CI 1.02–1.04), ethnicity (AOR 0.65, 95% CI 0.44–0.97), sex (AOR 1.50, 95% CI 1.02–2.22), family composition (AOR 1.62, 95% CI 1.13–2.31), and caring responsibility (AOR 0.52, 95% CI 0.30–0.90)

## Discussion

Adolescents with common mental health problems have difficulty transitioning from school to further education and employment and end up being ‘not in education, employment or training’ (NEET) as young adults (e.g., Witt et al., 2019). There is a significant care gap as the majority of young people do not access mental health services and receive early intervention (Soneson et al., 2022). Considering the long-term consequences of being NEET, a focus on secondary school students with common mental health problems is necessary before they leave compulsory education (Cornaglia et al., 2015, Soneson et al., 2022). Research on to what extent psychosocial factors are associated with later disengagement from education and employment is lacking for this population. This type of knowledge is needed to develop targeted preventive strategies during secondary schooling. In this study, it was possible to examine longitudinal associations between adolescent psychosocial factors and NEET status in young adulthood among a nationally representative sample of secondary school students with common mental health problems.

Main findings of this study were that having lower self-esteem, external locus of control, no job aspirations, and low to no physical activity significantly increased the likelihood of being NEET at ages 25–26 years whereas not being bullied and having more positive attitudes toward school decreased it for adolescents with common mental health problems. These findings remained significant even after controlling for socioeconomic, individual, and family characteristics in adolescence. Educational aspirations were significantly associated with later NEET status, a consistent finding with previous studies (Duckworth & Schoon, 2012), but its significance disappeared when other factors were taken into account suggesting that having more positive attitudes toward school is a better protective factor for avoiding NEET status although the magnitude is small. Moreover, early labour market engagement which reflects higher job aspirations appear to be a relatively stronger factor in order to avoid entering the NEET category. Previous studies have also emphasized that early job engagement is important for being employed later on (Baert et al., 2017) as well as to avoid becoming NEET (Duckworth & Schoon, 2012). In addition, one of the perceived barriers of service-seeking youth outside the labour force is indeed the lack of work experience (Ose & Jensen, 2017).

Previous studies show that behavioural problems are significantly associated with later NEET status; however, the current study shows that while this is true in the univariable model the association does not hold after adjustment for other factors whereas being bullied appears to be still important which is consistent with the literature (Tayfur et al., 2021). Similar results are found for self-evaluation factors including but not limited to self-esteem and locus of control, with both factors significantly associated with later NEET status regardless of background characteristics. Although the multiple imputation results for the multivariable model suggested that locus of control is not significant, the role of self-evaluations still appears to be important due to self-esteem. There are very few studies in the literature focusing on self-evaluation factors in the NEET context, but they indicate that low self-esteem and external locus of control are significantly associated with being disengaged from education and employment in young adulthood (Mendolia & Walker, 2015). Internal locus of control has been shown to protect disadvantaged youth against becoming NEET (Ng-Knight & Schoon, 2017). Moreover, one of the perceived barriers to education and employment among service-seeking youth is low self-esteem (Ose & Jensen, 2017).

In this study, physical activity was significantly associated with becoming disengaged from education and employment although this is relatively an overlooked factor in the literature. A recent systematic review has identified only one study for this domain (Tayfur et al., 2021). It is important to highlight that physical activity had the strongest magnitude of association with later NEET status over and above background characteristics. Participation in sport is recognized as a unique construct for positive psychosocial development (Bedard et al., 2020). It is important to focus on sport participation considering the benefits of physical activity for mental health as well as social competence of youth which can also elevate self-evaluations (Bedard et al., 2020). A recent study has showed that higher physical activity at age 15 is positively related to the likelihood of being employed in adulthood suggesting that investments in adolescent physical activity could yield better labour market outcomes (Kari et al., 2021). Therefore, sports may be an important supplemental component for mental health related interventions in schools to increase life chances. For instance, a recent innovation called the Game-Changer has been developed in Scotland to increase positive outcomes for vulnerable youth through sports demonstrating the potential for similar intervention strategies (Fitzpatrick et al., 2020).

There is an increasing interest in promoting healthy transitions into adulthood through school-based mental health interventions during adolescence (O’Connor et al., 2017). Considering the long-term consequences of being disengaged from education and employment, it is crucial to implement early strategies to prevent vulnerable youth entering the NEET category in the first place as the recurrence rate is also high (Arnold & Baker, 2013). As the findings of the current study supports it is preferable to do this during compulsory education given the difficulties of reaching young adults who are in NEET status because they are not attached to a singular institution. Moreover, interventions of re-engagement of NEET individuals and policy efforts have not been very successful (Hutchinson et al., 2016). It is also suggested that improvement in depressive symptoms among help-seeking young adults do not lead to re-engagement in employment or education (O’Dea et al., 2016). Therefore, preventive efforts should focus on other risk factors at play in order to deliver the most effective and targeted mental health interventions before adolescents leave the school setting.

Secondary schools are uniquely placed to identify mental health needs and provide care and support (Soneson et al., 2022). Given that many adolescents experience subclinical difficulties without receiving treatment, schools can deliver early interventions to equip adolescents with common mental health problems with the skills and competencies that they need to handle the subsequent transition period successfully (O’Connor et al., 2017, Soneson et al., 2022). School-based mental health provision in the UK is improving and one model of early identification of needs is through screening (Soneson et al., 2022). Utilizing any gain from early intervention is dependent upon screening to identify those who require it (Arnold & Baker, 2013). This study shows that prevention and promotion of mental health should have a focus on psychosocial factors. GHQ-12 is applicable in secondary school settings and can be used as a method of universal screening (screening *all* pupils) (Baksheev et al., 2011). However, relevant screening tools specific to NEET may be developed and implemented to further improve identification and preventive strategies. This may be used as a method of selective screening (screening *at-risk* pupils) among those with common mental health problems. It is suggested that more than half of those at risk for becoming NEET can be identified by age 14 (Arnold & Baker, 2013). Since interventions can be costly, targeted screening for NEET can identify those who will potentially benefit the most (Arnold & Baker, 2013). Therefore, development and implementation of cross-cultural screening tools should be encouraged such as the NEET- Hikikomori Risk Factors scale from Japan which also holds a psychosocial focus (Uchida & Norasakkunkit, 2015). To our knowledge, there is no such scale available in the UK. These developments should encompass a range of psychosocial factors, particularly the ones identified in this study. Mental health services may be supplemented by screening and fostering for a stronger trajectory of psychosocial wellbeing to encourage resiliency towards developmental tasks in the areas of work and education. Building on resiliency of students could help equip them with the assets to face the challenges that lie ahead once they leave the compulsory school setting and transition into adulthood (O’Connor et al., 2017).

The limitations of this study include that of secondary data analysis such as having no control over the data collected (Trzesniewski et al., 2011). Consequently, some of the factors are based on a single item which creates a limitation although efforts are made to ensure their effectiveness based on previous studies or indicators such as the Cronbach’s alpha. Moreover, this study could not include self-efficacy–another self-evaluation factor- as part of the interest of psychosocial factors despite its association with later education and employment status (Pinquart et al., 2003). The frequency of substance use also could not be included although it is suggested that the amount and frequency of use should be considered along with the use of different substances together when looking at longitudinal associations with education and employment (Tayfur et al., 2021). This may explain the current findings regarding substance use and should be considered in future research. Furthermore, both unemployed (without a job and actively seeking work within the last four weeks and are available to start work within the next two weeks) and inactive (without a job and not seeking work within the last four weeks and/or unable to start work in the next two weeks) young adults are considered to be NEET by definition nationally and internationally (Office for National Statistics, 2021, Eurofound, 2017). Therefore, an individual identified as NEET will always be either unemployed or economically inactive. Studies typically take the approach of classifying someone as NEET for whatever reason as long as they are not in any form of employment or education (e.g., Moore et al., 2015). So, unemployed and inactive subcategories such as looking after the home or disabled are grouped together (e.g., Roldós, 2014). However, the factors that predict NEET status may differ for those unemployed and those who are inactive. Therefore, it may provide further insight to examine this issue to better understand the heterogeneity within the group of young adults who are disengaged from education and employment. A study has drawn attention to this by distinguishing inactive NEETs from NEET by referring to them as ‘NLFET’ (neither in the labour force nor in education or training) and excluding unemployed youth because they are thought to be still a part of the labour force (Ose & Jensen, 2017). Unfortunately, the current study could not stratify analysis by subgroups of NEET due to the small number of participants in NEET status. However, this may be a good strategy to consider for future research.

## Conclusion

Research-based knowledge about the relationships between psychosocial factors and poor education and employment outcomes for adolescents with mental health problems is limited. This study identified the associations between adolescent psychosocial factors and later NEET status focusing on secondary school students with common mental health problems. The main findings were that lower self-esteem, external locus of control, low/no physical activity, and no job aspirations in adolescence were associated with an increased likelihood of being in NEET status as young adults whereas positive attitudes toward school and not being bullied were associated with a decreased likelihood for being in this status. This study showed that prevention and promotion of mental health should focus on psychosocial factors. School-based interventions during secondary education focusing on psychosocial factors, particularly physical activity could be helpful in this regard. Overall, these findings indicate that psychosocial factors play an important role in disengagement from education and employment after compulsory schooling for adolescents with common mental health problems. These study findings encourage provision of targeted mental health support at school, community, and clinical settings to prevent undesired educational and employment outcomes and improve life chances for at-risk youth.
